# Determinants of mortality among patients with drug-resistant tuberculosis in northern Nigeria

**DOI:** 10.1371/journal.pone.0225165

**Published:** 2019-11-19

**Authors:** Mamman Bajehson, Baba Maiyaki Musa, Mustapha Gidado, Bassey Nsa, Useni Sani, Ahmad T. Habibu, Ibrahim Aliyu, Tijjani Hussaini, AbdulRasheed Yusuf, Yakubu Gida

**Affiliations:** 1 KNCV/Challenge TB, Kano Regional Office, Kano, Nigeria; 2 Department of Medicine, Bayero University, Kano, Nigeria; 3 KNCV Tuberculosis Foundation, The Hague, Netherlands; 4 KNCV Nigeria/ Challenge TB, Country Office, Abuja, Nigeria; 5 Kano State Tuberculosis and Leprosy Control Program, Kano, Nigeria; 6 State Ministry of Health, Kano, Nigeria; 7 Katsina State Tuberculosis and Leprosy Control Program, Katsina, Nigeria; 8 Bauchi State Tuberculosis and Leprosy Control Program, Bauchi, Nigeria; Stellenbosch University, Desmond Tutu TB Centre, SOUTH AFRICA

## Abstract

**Background:**

Drug-Resistant tuberculosis (DR-TB) is estimated to cause about 10% of all TB related deaths. There is dearth of data on determinants of DR-TB mortality in Nigeria. Death among DR-TB treated cohorts in Nigeria from 2010 to 2013 was 30%, 29%, 15% and 13% respectively. Our objective was to identify factors affecting survival among DR-TB patients in northern Nigeria.

**Methods:**

Demographic and clinical data of all DR-TB patients enrolled in Kano, Katsina and Bauchi states of Nigeria between 1^st^ February 2015 and 30^th^ November 2016 was used. Survival analysis was done using Kaplan-Meier and multiple regression with Cox proportional hazard modeling.

**Results:**

Mean time to death during treatment is 19.2 weeks and 3.9 weeks among those awaiting treatment. Death was recorded among 38 of the 147 DR-TB patients assessed. HIV co-infection significantly increased probability of mortality, with an adjusted hazard ratio (aHR) of 2.35, 95% CI: 1.05–5.29, p = 0.038. Treatment delay showed significant negative association with survival (p = 0.000), not starting treatment significantly reduced probability of survival with an aHR of 7.98, 95% CI: 2.83–22.51, p = 0.000. Adjusted hazard ratios for patients started on treatment more than eight weeks after detection or within two to four weeks after detection, was beneficial though not statistically significant with respective p-values of 0.056 and 0.092. The model of care (facility vs. community-based) did not significantly influence survival.

**Conclusion:**

Both HIV co-infected DR-TB patients and DR-TB patients that fail to start treatment immediately after diagnosis are at significant risk of mortality. Our study showed no significant difference in mortality based on models of care. The study highlights the need to address programmatic and operational issues pertaining to treatment delays and strengthening DR-TB/HIV co-management as key strategies to reduce mortality.

## Introduction

Globally, tuberculosis (TB) is the ninth leading cause of death and leading cause from a single infectious agent, ranking above HIV/AIDS [1]. The proportion of people who develop TB and die from the disease (case fatality ratio for all forms of TB) worldwide, was 16% in 2016 with considerable country to country variation; from under 5% in a few countries to more than 20% in most countries in the WHO African region [1]. Drug-resistant TB (DR-TB) is the only major drug-resistant airborne infection and in 2016 was responsible for nearly one-third of deaths from antimicrobial resistance (AMR) [2]. Overall, DR-TB causes about 10% of all TB deaths [3] and is a threat globally. [4]

The World Health Organization (WHO) estimates the current proportion of patients with DR-TB as being 4.3% among new cases and 25% among previously treated cases in Nigeria [1]. Nigeria is among the 30 high burden countries for TB, multidrug resistant TB (MDR-TB) and TB/HIV. [1] The programmatic management of DR-TB (PMDT) in Nigeria commenced in 2010 with the program supporting care for all patients with isolates of any strain of Rifampicin-resistant TB (RR-TB), MDR-TB or extensively drug-resistant TB (XDR-TB).

In 2018, the country began full implementation of the shorter treatment regimen (STR) as the choice regimen for all eligible patients.

Globally, treatment success rate for MDR/RR-TB stands at 54% (2014 cohort). Nigeria’s treatment success rate of 74% for MDR/RR-TB (2014 cohort) [1] underscores the quality of the National TB and Leprosy Control Program. The treatment outcome data for death, though improving over the past successive cohorts, still remains sub optimal. It stood at 30%, 29%, 15% and 13% respectively for 2010, 2011, 2012 and 2013 (MDR/RR-TB cohorts) respectively. [Slide 10 in S1 Presentation]

There is paucity of data on factors associated with mortality in DR-TB patients in Nigeria. Consequently, this study aims to identify these factors.

## Materials and method

### Study and site design

This was a cross-sectional study on all enrolled DR-TB patients (age ranging from 10–80 years) in Kano, Katsina and Bauchi States. We reviewed records of 147 patients diagnosed between February 2015 and November 2016, including patients that had started treatment with the conventional regimen of second line TB drugs and those awaiting to start treatment for either confirmed MDR- or RR-TB. The GeneXpert mycobacterium tuberculosis rifampicin assay (Xpert MTB/ Rif assay) is the point of entry in the diagnosis of drug resistant tuberculosis in all of the three states in the study as is the National standard.

In each state, patients were allowed to choose their preferred mode of treatment (either facility-based or community-based) unless otherwise indicated on clinical and or programmatic grounds. All diagnosed RR-TB patients had baseline investigations which were reviewed prior to commencement of treatment. The conventional regimen consists of Kanamycin or Capreomycin, Levofloxacin, Cycloserine, Prothionamide and Pyrazinamide for 8 months intensive phase and less Kanamycin or Capreomycin for 12 months continuation phase. Pyridoxine was routinely provided daily.

### Inclusion and exclusion criteria

Only patients captured on the line list, an electronic data recording and reporting tool used by the program to track RR-TB patient detection and enrollment, were included in this review. All GeneXpert machines are equipped with a GxAlert system that automatically notifies through short message services, a minimum of four state level and two national level tuberculosis and leprosy control program personnel. Two of the state level personnel, one being the DR-TB focal person populates the line list, while the other, the state laboratory quality assurance officer, routinely contacts all GeneXpert facilities to both confirm and inquire about recent diagnosis. These personnel meet monthly to validate the line list at state level and same is replicated quarterly at regional level with personnel from the NTBLCP. Patients receiving treatment in facilities within the states but not resident in the any of the three states were excluded from this review. Patients either detected or commenced on treatment outside the period of review, even though resident in the states and on the line list, were also excluded from the review.

### Care protocol

All patients were managed in line with the National guidelines on PMDT. Each patient, upon testing positive on the GeneXpert MTB Rif assay as RR-TB, is notified to the State program and tracked into care to run baseline investigations and eventually placed on standardized conventional MDR-TB regimen. All patients are at different stages of management by their respective state TB and Leprosy Control Programs (STBLCP). Patients can belong to only one of two groups of care delivery, either facility based (which is a doctor driven care at specialized DR-TB treatment centers) or community based which is delivered at the homes of the patient by either a community nurse or community health extension worker.

Each patient is routinely monitored (clinically, bacteriologically and biochemically) a minimum of once a month for the entire duration of treatment and is given a monthly social support stipend by the program.

### Sampling and study procedures

Line listing of all diagnosed RR-TB cases from February 1^st^ 2015 to November 30^th^ 2016 per state was reviewed. Two out of the three states have an MDR-TB treatment facility (Kano and Bauchi) and all three states have the capacity to implement community-based care.

The primary outcome measure was death. Data on preselected variables such as: age, sex, HIV status, facility- or community-based initiation of therapy, duration from diagnosis to treatment were collected and entered into a spreadsheet.

### Statistical analysis

Data were cleaned for completeness and validation. Quantitative variables were presented in tabular form and summarized using mean and standard deviation. Categorical variables were also presented in tabular form and summarized using percentages.

Kaplan-Meier analysis was used to estimate the time to event. Predictors of mortality were investigated by cox proportional hazard regression. Variables associated with the outcome on univariate analysis defined as a P value <0.25 were included in a multivariate analysis, where a P value <0.05 was considered statistically significant. All statistical analysis were performed using SPSS 16 version 16.0 software.

### Ethics

The study was approved by the Health research ethics committee of Kano State Ministry of Health. Patient records used in the study is restricted to details contained in the anonymized line list. As this was a cross-sectional study of medical information collected during the course of routine clinical care, informed consent was waived by the committee.

## Results

### Patient demographics

Out of the 147 confirmed RR-TB cases, detected from 1^st^ February 2015 to 30^th^ November 2016, 45.5% were from Kano state, 32.6% from Katsina state and 21.7% from Bauchi state.

The median age was 33 years. Males accounted for 70.7% (104 patients) and 16.3% (24 patients) were HIV-positive [Table 1]. At the time of review for this study 38 patients (25.9%) had died [Table 1]. The mean age and standard deviation (SD) of those that died was 37.2 ± 12.3 years and 21 (55.3%) had commenced treatment prior to death.

**Table 1:** Patient demographics and key predictors (N = 147).

Demographics and key predictors	n (% of total)
Age	
10–15 years	6 (4.1)
16–24 years	24 (16.3)
25–34 years	48 (32.7)
35–44 years	42 (28.6)
45–54 years	16 (10.9)
55–80 years	11 (7.5)
Sex	
Male	104 (70.7)
Female	43 (29.3)
HIV Status	
Negative	123 (83.7)
Positive	24 (16.3)
Model Of Care	
Facility-based	55 (37.4)
Community-based	92 (62.6)
Died	
No	109 (74.1)
Yes	38 (25.9)

The highest proportion of patients in the study where within the young working class age group [Table 1].

Table 2 shows Katsina state as having only 8.3% of study patients being HIV co-infected and were able to initiate treatment in all 48 DR-TB patients. This study also demonstrated Katsina state as having the least percentage of mortality (18.8%) amongst the three states.

**Table 2:** Patient demographics segregated by States.

Demographics	n (% of total) by State		
	Kano	Katsina	Bauchi
Age			
10–15 years	5 (7.5)	0	1 (3.1)
16–24 years	14 (20.9)	6 (12.5)	4 (12.5)
25–34 years	17 (25.4)	22 (45.8)	9 (28.1)
35–44 years	21 (31.3)	12 (25)	9 (28.1)
45–54 years	5 (7.5)	4 (8.3)	7 (21.9)
55–80 years	5 (7.5)	4 (8.3)	2 (6.3)
Sex			
Male	47 (70.1)	35 (72.9)	22 (68.7)
Female	20 (29.9)	13 (27.1)	10 (31.3)
HIV Status			
Negative	53 (79.1)	44 (91.7)	26 (81.2)
Positive	14 (20.9)	4 (8.3)	6 (18.8)
Model Of Care			
Facility-based	15 (22.4)	10 (20.8)	30 (93.8)
Community-based	52 (77.6)	38 (79.2)	2 (6.2)
Died			
No	46 (68.7)	39 (81.2)	24 (75)
Yes	21 (31.3)	9 (18.8)	8 (25)

The predominant model of care in Bauchi State was facility based (93.8%) (Table 2) and had the highest percentage of patients initiated on treatment within 2 weeks of detection (40.6%). Kano and Katsina States had higher preference for community based model of care with patients initiated on treatment within 2–4 weeks of detection out performing initiation within 2 weeks of detection [Table 3].

**Table 3:** Detection to treatment initiation by State.

Duration from detection to treatment start	n (% of total) by State		
	Kano	Katsina	Bauchi
< 2 weeks	8 (11.9)	7 (14.6)	13 (40.6)
2–4 weeks	12 (17.9)	9 (18.8)	4 (12.5)
5–8 weeks	18 (26.9)	18 (37.5)	6 (18.8)
> 8 weeks	16 (23.9)	14 (29.2)	3 (9.4)
Did not start	13 (19.4)	0	6 (18.8)

Table 4 shows that HIV status (p = 0.000) in those DR-TB/HIV co-infected and on treatment had a significant association with death. Duration from detection to treatment start showed a significant association with survival (p = 0.000). Model of care differences, had no significant association with survival. [S1 Fig and S2 Fig]

**Table 4:** Data for predictors of survival using Kaplan-Meier (n = 38).

Variable		Dead (n = 38)			
		While on treatment (n = 21)	p-value	While awaiting treatment (n = 17)	p-value
			Log rank		Log rank
Time to event (death) in weeks	Mean + SD	19.2 + 13.9		3.9 + 3.2	
Sex	Male	15	0.959	11	0.21
	Female	6		6	
HIV status	Positive	3	0.000	10	0.846
	Negative	18		7	
Model of care	Facility based	7	0.286	9	0.496
	Community based	14		8	
Duration from detection to treatment start	<2 weeks	8	0.000	Not applicable	
	2–4 weeks	1			
	5–8 weeks	7			
	>8 weeks	5			

Results for the cox proportional model [Table 5] shows HIV co-infection as a variable that significantly increased the probability of mortality with an exponential coefficient of 2.35, 95% CI: 1.05–5.29, p = 0.038. Further breakdown for the hazard ratios associated with different times of treatment start using treatment initiation within 2 weeks from detection as our reference point, demonstrate an increased hazard of mortality, failing to start TB treatment increases the hazard of death 8 times compared to starting treatment within 2 weeks of detection. Starting treatment greater than 8 weeks after detection and within 2 to 4 weeks after detection slightly approaching significance with respective p-values of 0.056 and 0.092 is showed to have a lower hazard (beneficial).

**Table 5:** Determinants of mortality using Cox proportional hazard model (N = 147).

Covariate			Adjusted Hazard Ratio	95% CI	p-value
Age (N = 147)					
	Median	33 years	1.02	0.99–1.05	0.172
HIV Status					
HIV pos compared to HIV neg			2.35	1.05–5.29	0.038
Model of Care					
Community based compared to Facility based			1.37	0.65–2.88	0.404
Treatment delay					
Reference point: Treatment initiation within 2 weeks from detection					
	Did not start		7.98	2.83–22.51	0.000
	> 8 weeks		0.3	0.09–1.03	0.056
	5–8 weeks		0.44	0.15–1.34	0.150
	2–4 weeks		0.16	0.02–1.35	0.092

## Discussion

This study was embarked upon during a period the PMDT programs of Kano, Katsina and Bauchi states were recording high case fatalities beyond what we knew to expect as substantial increase in risk to mortality during treatment [5] from a drug-susceptible TB standpoint [6, 7]. The objective was to rapidly ascertain determinants of mortality among the patients who accessed health care and were tested.

Our data has shown an association between HIV and mortality in DR-TB patients. This observation is consistent with previous studies and keeps with the theme of rapidity of death with or without development of other opportunistic infections in the absence of effective treatment as noticed in HIV positive co-infected patients with MDR-TB [8–16].

The provision of effective treatment for patients infected with DR-TB is among the strongest actions in curbing further transmission of such strains [17, 18], our study showed that a large proportion of the patients that died were yet to be commenced on treatment.

A majority of the factors obstructing early commencement of treatment are programmatic delays that could be encountered on two fronts. The first occurs after diagnosis but before patient comes in contact with the program; this includes issues like poor access to same-day GeneXpert MTB/RIF assay result services, poor documentation of patients’ home or contact details on the laboratory sample request form, downtime of the GxAlert system leading to diagnosed cases remaining un-notified to the state program and lastly late notification of the Local Government TB and Leprosy Supervisor (LGTBLS) about the case notified from his/ her local government management unit. The second occurs once patients engage the program; this includes issues such as poor communication to patients on next steps upon disclosure of results, poor counseling of patients during disclosure, leading to patients denial of results or refusal to be enrolled into care, prolonged turnaround times for collation of baseline investigation results that are needed to inform subsequent management of patient and the occasional drug supply chain failures that result in stock-out of drugs required for initiation in the field.

Once patients engage the program further delays can arise from occasional prolonged turnaround times for release of results of patient baseline investigations.

The duration from detection to treatment start was also shown in this study to be a significant predictor of survival, with those who commenced within 2–4 weeks of detection having a better survival chances. Other studies have shown that starting appropriate treatment within four weeks of detection as having positive impact on survival [19] and this is particularly an important finding in our study as the current national guideline sets a quality benchmark of treatment commencement within two weeks of detection as the ideal for which all state programs should strive.

## Limitations

Even though the National and State Tuberculosis, Leprosy control programs in Nigeria continue to benefit from the technical support offered by funders through program implementing partners and the World Health Organisation; which ensures routine Tuberculosis data recording and reporting is at a high level of quality, there are important limitations to consider in this study. First, we did not have a method to assess the state of each patient at presentation either through clinical notes/records at enrollment or baseline laboratory parameters at enrollment. However, based on the existing national guidelines for PMDT in Nigeria during that period (2015 edition), existing criteria for exclusion from community-based model of care would mean those patients presenting with any evidence of advanced disease or heightened possibility of unfavorable outcome will be managed using the facility-based model.

Second, the mortalities reported in this study may differ from those eventually reported as final outcome for these 147 patients in their respective cohorts as final outcomes are reported 2–3 years after close of enrollment of the annual cohort.

Third, variability in skill, training and cadre of health care worker managing patients either in the facility or community may have introduced an unquantifiable bias. Generally in facilities, a team (medical officer, nurse, pharmacist and laboratory scientist) are involved in case management, while in the community-based model the majority of service providers are from the community such as health extension workers or community health officer cadres and to a lesser extent nurses.

## Conclusion

The HIV co-infected DR-TB patient and DR-TB patients that have been diagnosed but are not placed on treatment, are at a significant risk of mortality.

Compromise in DR-TB/HIV collaboration activities in PMDT could worsen mortality among this important sub-population.

Our finding shows that the model of care (facility- vs. community-based), did not significantly influence survival. Similar studies have shown that quality-assured community-based PMDT is possible and would reduce both costs and nosocomial spread of DR-TB [20, 21]. This eliminates the earlier fears of decentralization of PMDT services and justifies the nation’s current drive to scale up community enrollment.

Shortening the time between detection and initiation on appropriate treatment is validated by other studies [20] and for the quality benchmark of treatment initiation within two weeks of detection to be upheld in Northern Nigeria, more studies on timing of treatment initiation for DR-TB patients in our setting is required.

As Nigeria adopts the shorter treatment regimen (STR), which is a more potent regimen associated with better treatment outcomes [22] our work emphasizes the need to address programmatic and operational issues that cause delays with patient treatment initiation while strengthening DR-TB/HIV co-management as key strategies in avoiding poor outcomes.

## Supporting information

S1 Presentation National tuberculosis and leprosy control program performance update 2017. (PDF)

S1 Fig Survival functions by HIV status. (TIF)

S2 Fig Survival functions by duration from detection to treatment start. (TIF)
