# Supplementary material for: Determinants of mortality among patients with drug-resistant tuberculosis in northern Nigeria
Source: PLoS One. 2019 Nov 19;14(11):e0225165. doi: 10.1371/journal.pone.0225165 (PMC6863558; doi:10.1371/journal.pone.0225165)
Supplement: S1 Presentation — (PDF) [file pone.0225165.s001.pdf]

PROGRAMME PERFORMANCE UPDATE FOR THE 2017 PROGRAMME REVIEW MEETING

BY
Dr. Simeon Onyemaechi
on behalf of the
NATIONAL COORDINATOR, NTBLCP, FMoH

GIGINYA HOTEL, SOKOTO, SOKOTO STATE

28/11/2017

DATE: 28TH NOVEMBER, 2017

FEDERAL MINISTRY OF HEALTH

PRESENTATION OUTLINE

- Programme Background
- Key Indicators for 2016
- Key Achievements
- Current challenges
- Way Forward

FEDERAL MINISTRY OF HEALTH

INTRODUCTION

- The National Tuberculosis and Leprosy control programme (NTBLCP) was established in 1988 but Launched in 1991
- Mandate is to ensure the control of Tuberculosis, Leprosy and Buruli ulcer in Nigeria
- Operates along the three tiers of Government
- The NTBLCP currently operates a 6 year National Strategic Plan (NSP) for Tuberculosis (2015 – 2020) and a 5 year strategic plan for both Leprosy and BU (2016 – 2020)

FEDERAL MINISTRY OF HEALTH

VISION AND GOAL OF THE NTBLCP

Vision:

A Nigeria free of TB, Leprosy and Buruli Ulcer

Goal:

The overall goal is to reduce significantly the burden, socio-economic impact, and transmission of TB, Leprosy and Buruli ulcer in Nigeria in line with the SDG and the WHO End TB strategy.

FEDERAL MINISTRY OF HEALTH

TARGETS FOR TB CONTROL IN NIGERIA

Goal of the National Strategic Plan for TB 2015 – 2020:

- Ensure universal access to high-quality, patient-centred TB prevention, diagnosis and treatment services for Nigerians with all forms of TB.

Impact indicators and targets:

- To decrease prevalence rate of TB from 326/100,000 population in 2013 to 163/100,000 in 2025

- To decrease TB mortality rate from 94/100,000 population in 2013 to 24/100,000 in 2025

TARGETS FOR LEPROSY AND BURULI ULCER CONTROL IN NIGERIA

Goal of the National Strategic Plan for Leprosy and BU 2016 – 2020:

- Reduction of new Grade 2 Disability cases to less than one case per million population by 2020
- Zero Grade 2 disabilities among new child cases by 2020
- Elimination of leprosy as a public health problem at sub-national level by 2020

Impact indicators and targets:

- Reduction of the proportion of category lesion 3 less than 50%
- The proportion of ulcerative lesions at diagnosis reported to be less than 60%
- The proportion of patients presenting with limitation of movement at diagnosis to be below 15%
- At least 70% of cases reported to be confirmed by PCR

FEDERAL MINISTRY OF HEALTH

TB SITUATION IN NIGERIA ...1

2017 Global TB report for Nigeria revealed that:

- Nigeria is included in the 30 high burden countries for TB, TB/HIV and MDR-TB.
- Ranked 7th among the 30 high TB burden countries and 2nd in Africa
- Accounts for 8% of the global gap between TB incidence and notified cases.
- TB treatment coverage 24%
- The incidence of all forms of TB is 219/100,000 population

FEDERAL MINISTRY OF HEALTH

TB SITUATION IN NIGERIA…2

- Mortality rate (excluding HIV + TB) for all forms of TB is 62/100,000 population

- Mortality rate (HIV + TB only) for all forms of TB is 21/100,000 population

- 94% of TB patients know their HIV status.

- Proportion of notified HIV-positive TB cases receiving ART is 83%.

FEDERAL MINISTRY OF HEALTH

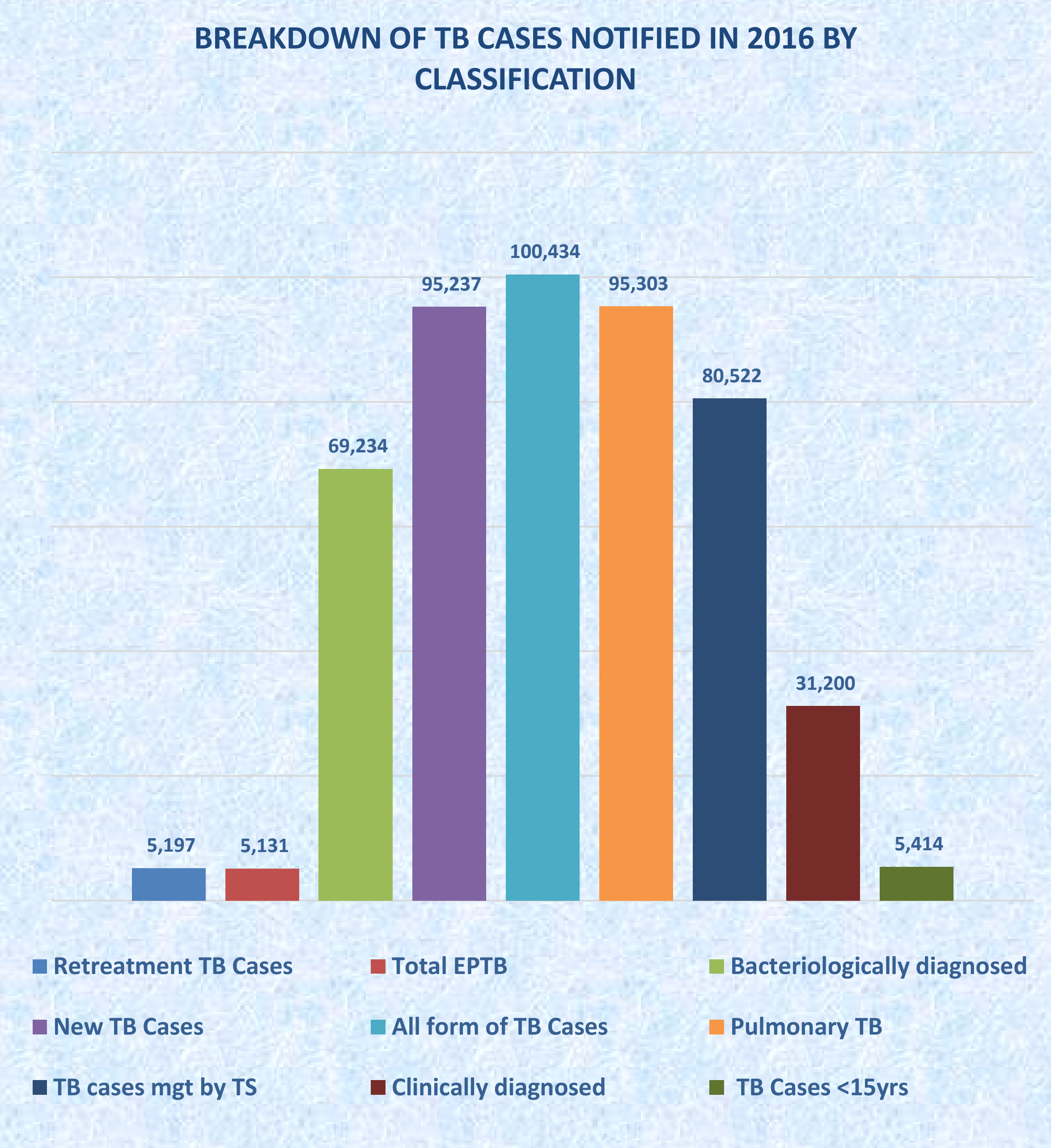
BREAKDOWN OF TB CASES NOTIFIED IN 2016 BY CLASSIFICATION
5,197
5,131
69,234
95,237
100,434
95,303
80,522
31,200
5,414
Retreatment TB Cases
Total EPTB
Bacteriologically diagnosed
New TB Cases
All form of TB Cases
Pulmonary TB
TB cases mgt by TS
Clinically diagnosed
TB Cases <15yrs

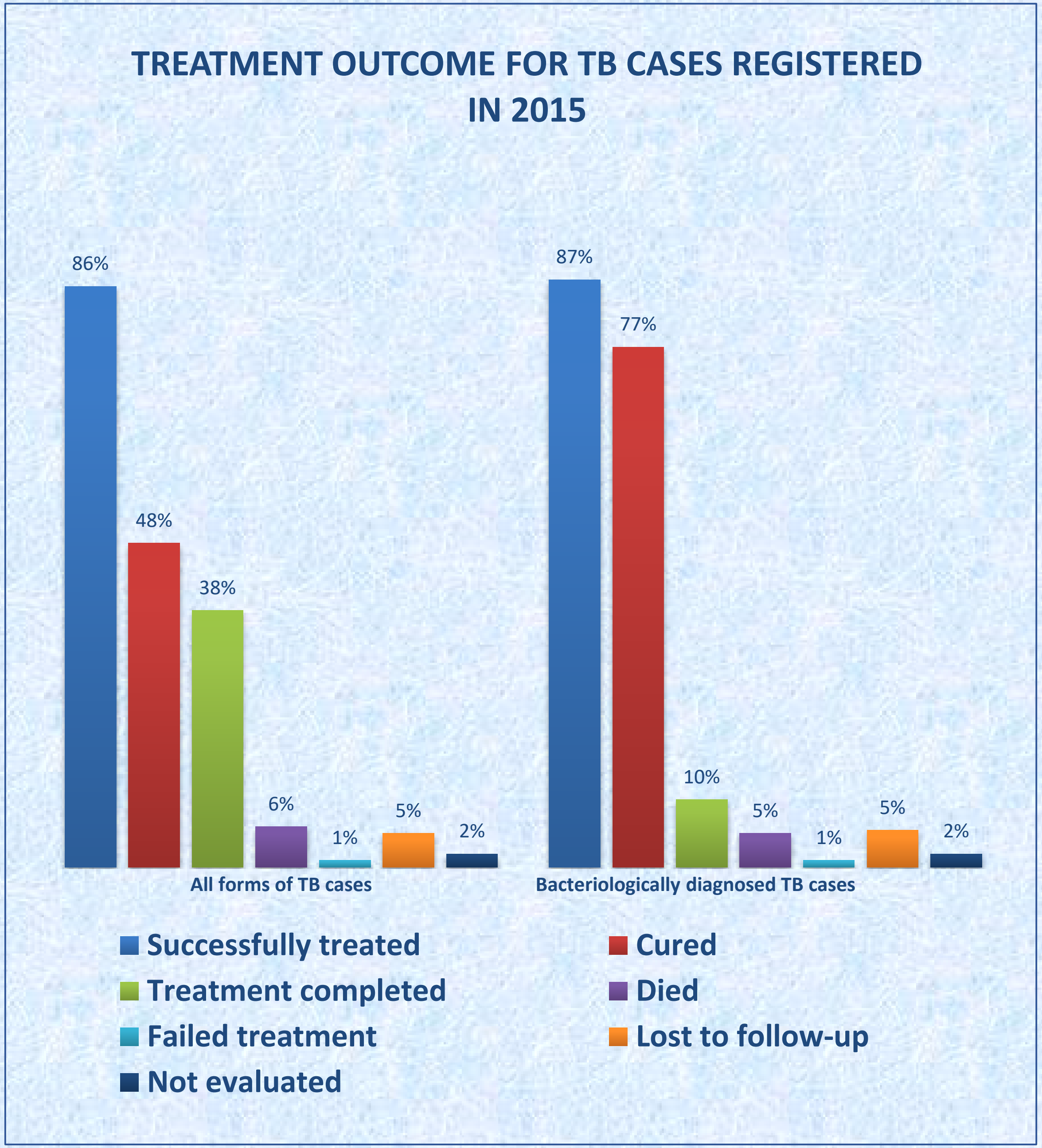
TREATMENT OUTCOME FOR TB CASES REGISTERED IN 2015
86%
48%
38%
6%
1%
5%
2%
87%
77%
10%
5%
1%
5%
2%
All forms of TB cases
Bacteriologically diagnosed TB cases
Successfully treated
Cured
Treatment completed
Died
Failed treatment
Lost to follow-up
Not evaluated

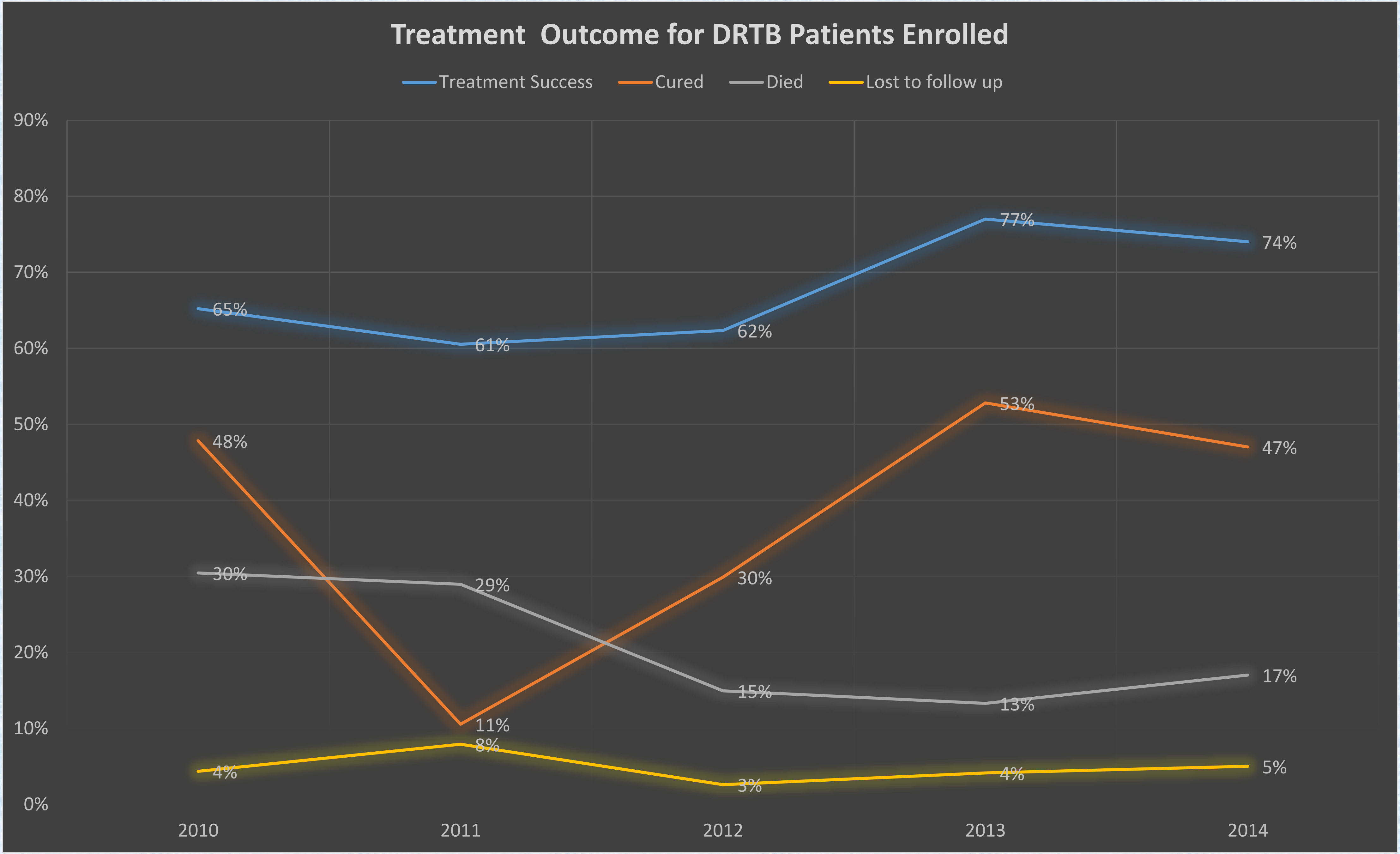
Treatment Outcome for DRTB Patients Enrolled
Treatment Success
Cured
Died
Lost to follow up
90%
80%
70%
60%
50%
40%
30%
20%
10%
0%
2010
2011
2012
2013
2014
65%
61%
62%
77%
74%
48%
11%
30%
53%
47%
30%
29%
15%
13%
17%
4%
8%
3%
4%
5%

Key achievements

- Launching of WOW project by the HMH for accelerated TB case finding
- Key documents for introduction of new paediatric formulation developed

i. Rapid advice on new paediatric formulation

ii. Orientation manual for healthcare providers on new paediatric formulation

iii. SOPs/IEC materials on new paediatric formulation

- Training of trainers on the new paediatric formulation

- Robust collaboration with Paediatric Association of Nigeria/NISPID for childhood TB case finding

Key achievements Contd.

- Conducted programmatic gap analysis and epidemiological analysis
- Conducted surveys on the quality care for TB patients and catastrophic cost suffered by TB patients
- Successful roll out of the national electronic TB information management system to twenty nine plus one states of the federation and 322 high burden sites, with the remaining seven states to be completed before the end of the year
- Successful participation in the GDF GLC mission in Nigeria

Challenges

- Dwindling fund for TB control activities (both government and donor)

- Low TB case finding (Adult and children)

- Sub-optimal reporting on the national electronic TB information management system by all state programs

WAYFORWARD

- Strategic engagement of governments at all levels to provide adequate funding for the TBL & BU control programme
- Adopt innovative ways to improve TB case finding
- Optimize the national electronic TB information management system to help improve data management
- Immediate adoption of the shorter drug regimen for DRTB patients to improve outcome and reduce side effects

FEDERAL MINISTRY OF HEALTH

THANK YOU FOR LISTENING

FEDERAL MINISTRY OF HEALTH
