# Supplementary figures and images for: Determinants of mortality among patients with drug-resistant tuberculosis in northern Nigeria
Source: PLoS One. 2019 Nov 19;14(11):e0225165. doi: 10.1371/journal.pone.0225165 (PMC6863558; doi:10.1371/journal.pone.0225165)

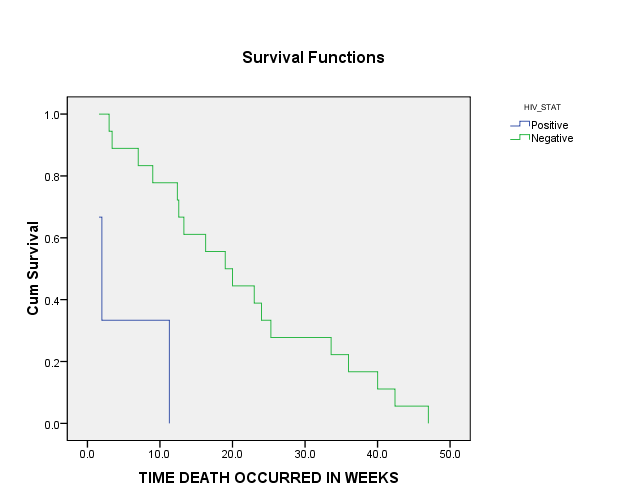

Supplement: S1 Fig — (TIF) [file pone.0225165.s002.tif]

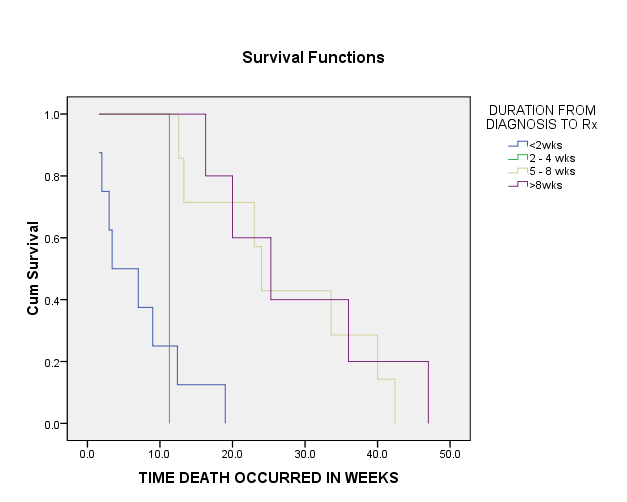

Supplement: S2 Fig — (TIF) [file pone.0225165.s003.tif]
